# A study on the relationship between waist phenotype, hypertriglyceridemia, coronary artery lesions and serum free fatty acids in adult and elderly patients with coronary diseases

**DOI:** 10.1186/s12979-018-0119-6

**Published:** 2018-06-15

**Authors:** Rui-Feng Yang, Hanlei Zhang, Zhongchun Wang, Xiao-Yong Liu, Zhi Lin

**Affiliations:** 1 Department of Cardiology, the 2nd Hospital Affiliated with Kunming Medical University, Kunming, 650101 Yunnan Province China; 2 Yunnan Health Education Institute, Kunming, 650000 Yunnan Province China; 3 Hospital of Yimen Country, Yimen, 651100 Yunnan Province China

**Keywords:** Coronary disease, Hypertriglyceridemia waist phenotype, Waist circumference, Free fatty acid

## Abstract

**Background:**

Abdominal obesity is an independent risk factor for coronary heart disease (CHD) and high serum triglyceride (TG) and free fatty acid levels may precipitate or aggravate CHD.

**Methods:**

We enrolled patients with coronary heart disease in our hospital from October 2008 to July 2009. Patients with high TG and increased WC, i.e. waist phenotype WP were included in group A. In group B, were included patients with high TG but not WP. Group C consisted of patients with WP but not high TG. Finally, Group D was composed of patients without high TG or WP. Serum FFA levels for all patients were measured by ELISA. The relationship between TG levels, WC, FFA levels, and coronary artery score was analysed by a single variable regression.

**Results:**

Group A had a significantly higher FFA level than the other groups. Regression analysis showed that FFA, TG, WC, hip circumference, waist-to-height ratio, systolic blood pressure, pulse pressure index, and low-density lipoprotein cholesterol all positively correlated with CAS (*r* = 0.160 ~ 0.415, *P* = 0.000 ~ 0.032). After we controlled for traditional risk factors for cardiovascular disease, FFA levels remained positively correlated to the CAS (*r* = 0.365, *P* < 0.001).

**Conclusion:**

The serum FFA level for patients with complications of both increased WC and high TG levels was significantly higher than that of patients without either of these complications. The close correlation between the CAS and FFA levels showed by regression analysis suggested that inflammation in these patients was more serious. Increased WC and high TG levels as well as FFA level are valuable for the prediction of cardiovascular disease and can be applied as a clinical guidance for early intervention in the treatment of coronary heart diseases.

## Background

Atherosclerosis is a pathological progress in which the development and formation of the atherosclerotic plaque is closely linked to increased inflammation [1, 2]. Although coronary heart disease (CHD) mortality rates have declined over the past four decades in western countries, this condition remains responsible for one-third of all deaths in individuals over age 35 [3]. Serum free fatty acids (FFA) play an important role in the occurrence and development of CHD, since they can lead to oxidative stress, trigger inflammatory responses and cause abnormal vascular reactivity [4]. In fact, in this last study, the authors have shown that an increase in plasma FFA concentrations acutely causes, in circulating peripheral blood cells, an increase in the intranuclear NF-κB binding activity, a cardinal step in the induction of inflammation, as well as reactive oxygen species generation by these cells. In addition, they demonstrated that an increase in FFA also induces an increase in macrophage migration inhibitory factor, a pro-inflammatory cytokine secreted by monocytes and adrenocorticotrophs in the pituitary gland [4]. These data underscore the importance of monitoring FFA levels for the prevention of CHD.

In the clinic, the levels of several factors in the lipid profile of patients with CHD are closely monitored. These factors are low-density lipoprotein cholesterol (LDL-C), high-density lipoprotein cholesterol (HDL-C) and total cholesterol (TC). In addition to these factors, there are also factors that are regarded as residual risks, namely, triglycerides (TG) and non-high-density lipoprotein cholesterol [5]. In this last study, the most noteworthy finding was the reduced risk of CHD with low on-treatment TG (< 150 mg/dl) that was independent on the level of LDL-C. For each 10-mg/dl decline in on-treatment TG, the authors observed a 1.6% lower risk of the composite end point after adjustment for LDL-C and other covariates. Moreover, the combination of low LDL-C (< 70 mg/dl) and low TG (< 150 mg/dl) was associated with the lowest event rates compared with higher LDL-C, higher TG, or both. Thus, on-treatment TG < 150 mg/dl was independently associated with a lower risk of recurrent CHD events, lending support to the concept that achieving low TG may be an additional consideration beyond low LDL-C in these patients [5].

Abdominal obesity is an independent risk factor for CHD [6, 7]. CHD patients with high TG levels and aberrant waist circumference (WC) may or may not exhibit increased inflammatory response. In this study, we measured serum FFA levels in the CHD patients with different WC and different TG levels. We also analysed the correlation between FFA and the coronary artery score (CAS). Our aim was to further explore the correlation of hypertriglyceridemia and increased WC, i.e. waist phenotype (WP) and FFA with CHD.

## Methods

### Patients

Study participants were selected from patients who visited the Department of Cardiology from October 2008 to July 2009. A total of 52 CHD patients with an average age 65 ± 6 (39 males and 13 females) with high TG and WP were included in group A. In group B, 38 CHD patients with mean age 62 ± 10 years (34 males and 4 female) with high TG but not WP were included. Group C consisted of 45 CHD patients (38 males and 7 females) with an average age 61 ± 11 years with WP but not high TG. Finally, Group D was composed of 59 CHD patients with a mean age 66 ± 9 years without high TG or WP. The definition of high TG for both males and females was established as a TG level greater than 1.70 mmol / L, and the definition of WP for males was a WC ≥ 90 cm and for females a WC ≥ 85 cm. We followed the CHD diagnosis standards outlined in the guidelines established by the Chinese Society of Cardiology in 2001 and 2007 [8]. All patients were confirmed by coronary angiography to have stenosis ≥50% in one or more of the coronary arteries [9]. CHD diagnostic criteria also included chronic stable angina (including asymptomatic coronary artery disease and cardiac syndrome X) and acute coronary syndrome. All those who had the following conditions or who underwent the listed treatments were excluded: secondary hyperlipidaemia, severe cardiac dysfunction, valvular heart disease, cardiomyopathy, liver or kidney disease, inflammatory disease, acute or chronic infection, acute infectious disease, recent surgery or trauma, abnormality in blood or immune system, cancer or immunosuppressive therapy.

### Basic data collection

Patients were fasted for 12 h during the period from dinner to the morning of the study. Blood samples were taken and tested for blood glucose levels and the lipid profile including LDL-C, HDL-C and TG (measurement units are mmol/L). The LDL-C and HDL-C testing kits were purchased from Wako Diagnostics (Japan). TG testing was carried out using the GPO-POD method, a kit from Shanghai Kehua Bio-engineering Co., Ltd. Fasting plasma glucose was determined by the glucose oxidase method. For WC measurements, fasted patients were measured while standing erect with the abdominal muscles relaxed and feet placed apart a distance of 25 to 30 cm; the measuring tape was placed horizontally at the midpoint of the lowest rib and the iliac crest and the measurement was taken at the end of the expiration. Hip circumference (HC) measurement was taken when the WC was taken: the tape was placed horizontally at the pubic symphysis and the largest circumference was recorded with an accuracy of ±1 cm. Waist-hip ratio (WHR) calculation used was WHR = WC (cm) / HC (cm); Waist-to-height ratio (WHtR) calculation used was WHtR = WC (cm) / height (cm); body mass index (BMI) calculation used was BMI = weight (kg) / height^2^ (m^2^). All the data measured were calculated twice with computers. Blood pressure measurements taken were systolic blood pressure (SBP), diastolic blood pressure, mean arterial blood pressure, pulse pressure (PP) and pulse pressure index (PPI). All blood pressure indices were measured in mmHg except the PPI. Arterial blood pressures were measured during hospitalization and the average was taken. Coronary angiography was performed using the GE Advantx single C-arm. The conventional Judkin method was used to image the left and right coronary arteries selectively. Other positioning methods were also considered as necessary. For the CAS calculation, a positioning that can show the severity of a single lesion the most was selected and a quantitative computer analysis system was utilized to determine the degree of coronary artery stenosis. The CAS was calculated by the Leaman’s method [10].

### FFA testing

The double-antibody sandwich ABC-ELISA method (Yantuo, Shanghai, China) was used to measure FFA levels.

### Statistical analysis

All statistical analysis was performed on the SPPS 13.0 software. Count data were analysed using the chi-square (χ^2^) test. The quantitative data were presented as mean ± standard deviation. Group means were compared using analysis of variance (ANOVA). Single variable regression analysis (Pearson’s method) was performed to analyse the correlation between the CAS (Leaman’s score) and various cardiovascular risk factors. *P* < 0.05 was considered statistically significant.

## Results

### Comparison of basic data and physical indices

There was no statistically significant difference in gender or age amongst the four groups. There was a difference in WC means between group A and B (*P* < 0.05), A and D (*P* < 0.05), B and C (*P* < 0.05), B and D (*P* < 0.05), and C and D (*P* < 0.05). The BMI, WHR and WHtR of A, C, B and D decreased gradually. There was no significant difference between the groups for BMI or WHR. However, significant differences (*P* < 0.05) in WHtR were observed between group A and B, A and D, B and C, C and D (Table 1). No significant differences were found in the blood pressure values between all these groups (data not shown).

**Table 1:** Comparison of basic data and physical indices

Indices	Group A (*n* = 52)	Group B (*n* = 38)	Group C (*n* = 45)	Group D (*n* = 45)
Sex (male/female)	39/13	34/4	38/7	39/6
Age	61.63 ± 11.373	61.66 ± 10.435	61.16 ± 11.691	66.00 ± 9.325
WC(cm)	94.95 ± 5.843	85.55 ± 6.500	95.38 ± 5.558	83.03 ± 5.607
HC(cm)	99.56 ± 7.09	90.29 ± 4.738	99.94 ± 6.185	89.89 ± 4.343
WHR^a^	0.97 ± 0.045	0.94 ± 0.046	0.96 ± 0.047	0.92 ± 0.050
WHtR^b^	0.58 ± 0.037	0.51 ± 0.028	0.57 ± 0.041	0.50 ± 0.034
BMI^c^	26.39 ± 3.273	23.50 ± 2.503	25.88 ± 2.121	22.61 ± 2.331

^a^Waist-hip ratio = WC (cm) / HC (cm)

^b^Waist-to-height ratio = WC (cm) / Height (cm)

^c^BMI=Weight (kg) /Height^2^ (m^2^)

### Comparison of blood glucose and lipid profiles

The TC level of group B was significantly higher than those of the other three groups (*P* < 0.05). There was no significant difference between the TC levels of groups A, C and D. The TG level of group B was significantly higher (*P* < 0.05) than the other three groups and the TG level of group A was significantly higher than those of group C and D (*P* < 0.05). The mean TG levels of group C and D were not significantly different. The HDL-C level of group B was significantly lower than the other three groups (*P* < 0.05). There was no significant difference between groups A, C and D. Levels of LDL-C and FPG were not significantly different between the groups (Table 2).

**Table 2:** Comparison of blood glucose and lipid profiles

Indices	Group A (*n* = 52)	Group B (*n* = 38)	Group C (*n* = 45)	Group D (*n* = 45)
TC (mmol/L)	4.65 ± 1.064	4.91 ± 1.084	4.48 ± 1.296	4.21 ± 0.976
TG (mmol/L)	2.98 ± 1.195	6.66 ± 4.400	1.22 ± 0.309	1.10 ± 0.305
HDL-C(mmol/L)	1.00 ± 0.607	0.87 ± 0.268	1.04 ± 0.310	1.14 ± 0.334
LDL-C (mmol/L)	2.89 ± 0.886	2.98 ± 0.842	2.80 ± 1.203	2.57 ± 0.897
FPG (mmol/L)	5.44 ± 1.785	5.71 ± 2.102	6.18 ± 2.544	5.33 ± 1.424

### Comparison of the FFA and CAS

Group A had a significantly higher (*P* < 0.05) FFA level compared to the other three groups. The FFA level of group B and C were not significant different. Both group B and C had significantly higher (*P* < 0.05) FFA levels than group D. The CAS of group A, B and C were not significantly different although there appeared to be a trend towards decreased CAS (group A > group C > group B > group D) (Table 3).

**Table 3:** Comparison of the FFA and CAS

Indices	Group A (*n* = 52)	Group B (*n* = 38)	Group C (*n* = 45)	Group D (*n* = 45)
FFA (mmol/L)	121.81 ± 83.965	73.16 ± 55.668	79.59 ± 45.334	43.94 ± 25.192
CAS	10.47 ± 7.459	7.50 ± 6.121	8.20 ± 5.405	5.47 ± 4.123

### Single variable regression analysis: CAS as the dependent variable

The CAS was used as the dependent variable and cardiovascular disease risk factors and inflammatory indices were set as independent variables. The regression showed that FFA, TG, WC, HC, WHtR, SBP, PPI and LDL positively correlated with the CAS (*P* = 0.012) (Table 4). The rest of the factors and indices did not significantly correlate with the CAS (*r* = − 0.073 ~ 0.135, *P* = 0.241 ~ 0.719). After we controlled for traditional risk factors of cardiovascular disease, FFA levels remained positively correlated with the CAS (*r* = 0.365, *P* < 0.001).

**Table 4:** Regression of the CAS on the cardiovascular risk factors and inflammatory indices

Indices	Correlation coefficient (*r*)	*P* value
FFA	0.415	0.000
TG	0.204	0.006
HC	0.169	0.019
WHtR	0.184	0.014
SBP	0.160	0.032
PP	0.250	0.005
PPI	0.219	0.003
LDL-C	0.187	0.012

### Single variable regression analysis: FFA as the dependent variable

We established a regression of FFA against other cardiovascular risk factors and found that TG, WC, HC, BMI, WHtR and WHR all positively correlated with FFA. The remaining factors did not significantly correlate with FFA. (*r* = − 0.044 ~ 0.085, *P* = 0.074 ~ 0.975) (Table 5).

**Table 5:** Regression of the FFA on the physical indices

Indices	Correlation coefficient (*r*)	*P* value
TG	0.174	0.020
WC	0.235	0.002
HC	0.169	0.025
WHR	0.166	0.027
WHtR	0.242	0.001

## Discussion

Abdominal fat can be distributed in two ways. Fat can deposit beneath the skin (subcutaneous fat) or accumulate in the abdominal cavity, which is termed organ fat or visceral fat. The latter fat deposit type carries more clinical implications. The WC measurement can be used as a rough estimation of this visceral adipose accumulation. Although obesity can undermine the health condition, excess fat does not necessarily lead to metabolic abnormalities [11]. Studies have shown that increase in small, dense LDL-C caused by high TG levels is a crucial contributor to atherosclerosis and can promote atherosclerotic plaque rupture [12]. Therefore, fasting TG level is an indirect reflection of LDL-C level and high TG hyperlipidaemia is a risk factor for CHD. It was pointed out that high TG level increased the risk of cardiovascular diseases by 30% in men and approximately 75% in women [13]. It was also reported that for each l mmol / L increase in plasma TG, the risk for CHD increased by 36% in male and by 70% in women [14]. It has been observed that compared with an individual with a normal WC and TG level, a subject with high TG and WP has higher blood pressure, increased apolipoprotein B and C, lower HDL-C and apolipoprotein A-I levels and lower small LDL [15]. Additionally, high TG and WP increased the risk of cardiovascular disease [16]. A high TG level (1.45 mmol / L) combined with an increased WC (88 cm) in menopausal women may be a good indicator for cardiovascular disease [17]. Thus, increased WC and an elevated TG level could be used as effective markers to predict CHD risk.

A disruption of cardiac energy metabolism is often seen in patients with CHD. Coronary atherosclerosis frequently leads to myocardial ischemia followed subsequent hypoxia and impaired myocardial energy metabolism. One of the hallmarks of this metabolic imbalance is blocked β-oxidation of fatty acids, resulting from reduced transport of FFA into the mitochondria. Reduced fatty acid oxidation causes the accumulation of harmful intermediates such as fatty acyl-CoA and acyl carnitine in the cytosol and mitochondria, which will inhibit acyl-CoA synthetase and decrease FFA uptake, ultimately leading fatty acid levels in the cardiac muscle to quickly rise. Myocardial ischemia and hypoxia also excite the sympathetic nerve, which triggers the release of FFA from the adipose tissue via β-receptors and elevates the serum FFA and eventually intramyocardial FFA [18]. Hence, serum FFA levels are related to the degree of myocardial ischemia and hypoxia [19, 20]. We found that CAS is positively correlated with serum FFA levels, which suggests that FFA may promote coronary atherosclerosis formation and development. Based on our findings, it is suggested that FFA is a new, effective marker of risk and also a new therapeutic target for cardiovascular disease.

The FFA level is also relevant to symptoms of metabolic syndrome such as abdominal obesity and high TG hyperlipidaemia [21]. In obesity, adipose cells increase in volume, total body fat increases and the release of fatty acids also increases accordingly. The dynamic equilibrium of the release and consumption of FFA disappears, and serum FFA level is augmented. We found a significant, positive correlation between WC, HC, BMI, WHtR, WHR and FFA levels. We also found that FFA levels for subjects with both high TG and WP were significantly greater than those with just either high TG levels or WP and those with neither of these two conditions.

## Conclusion

The findings of our study indicate that CHD patients with high TG hyperlipidaemia as well as WP had a higher serum FFA, suggesting that these patients suffer more severe inflammation. Patients who have both of high TG and WP are more susceptible to coronary atherosclerosis and therefore require specialized care and adequate monitoring of symptoms. Medications and changes in lifestyle should be prescribed to reduce the level of TG and WC, which will reduce residual risk and occurrence of cardiovascular events and mortality. However, pharmaceutical approaches developed for the treatment of obesity, despite short-term benefits, often are associated with rebound weight gain after the cessation of drug use and serious side effects deriving from the medication can occur. Thus, nutraceutical interventions have been proposed as a new opportunity in obesity treatment [22].

## Abbreviations

BMI: Body mass index
CAS: Coronary artery score
CHD: Coronary heart disease
FFA: Serum free fatty acids
HC: Hip circumference
HDL-C: High-density lipoprotein cholesterol
LDL-C: Low-density lipoprotein cholesterol
PP: Pulse pressure
PPI: Pulse pressure index
SBP: Systolic blood pressure
TC: Total cholesterol
TG: Triglycerides
WC: Waist circumference
WHR: Waist-hip ratio
WHtR: Waist-to-height ratio
WP: Waist phenotype
